# FAST (fast analytical simulator of tracer)-PET: an accurate and efficient PET analytical simulation tool

**DOI:** 10.1088/1361-6560/ad6743

**Published:** 2024-08-06

**Authors:** Suya Li, Mahdjoub Hamdi, Kaushik Dutta, Tyler J Fraum, Jingqin Luo, Richard Laforest, Kooresh I Shoghi

**Affiliations:** 1 Mallinckrodt Institute of Radiology, Washington University School of Medicine, St Louis, MO, United States of America; 2 Imaging Science Program, McKelvey School of Engineering, Washington University in St Louis, St Louis, MO, United States of America; 3 Department of Surgery, Washington University School of Medicine, St Louis, MO, United States of America; 4 Department of Biomedical Engineering, Washington University in St Louis, St Louis, MO, United States of America

**Keywords:** positron emission tomography, analytical simulation, GATE simulation

## Abstract

*Objective.* Simulation of positron emission tomography (PET) images is an essential tool in the development and validation of quantitative imaging workflows and advanced image processing pipelines. Existing Monte Carlo or analytical PET simulators often compromise on either efficiency or accuracy. We aim to develop and validate fast analytical simulator of tracer (FAST)-PET, a novel analytical framework, to simulate PET images accurately and efficiently. *Approach*. FAST-PET simulates PET images by performing precise forward projection, scatter, and random estimation that match the scanner geometry and statistics. Although the same process should be applicable to other scanner models, we focus on the Siemens Biograph Vision-600 in this work. Calibration and validation of FAST-PET were performed through comparison with an experimental scan of a National Electrical Manufacturers Association (NEMA) Image Quality (IQ) phantom. Further validation was conducted between FAST-PET and Geant4 Application for Tomographic Emission (GATE) quantitatively in clinical image simulations in terms of intensity-based and texture-based features and task-based tumor segmentation. *Main results.* According to the NEMA IQ phantom simulation, FAST-PET’s simulated images exhibited partial volume effects and noise levels comparable to experimental images, with a relative bias of the recovery coefficient RC within 10% for all spheres and a coefficient of variation for the background region within 6% across various acquisition times. FAST-PET generated clinical PET images exhibit high quantitative accuracy and texture comparable to GATE (correlation coefficients of all features over 0.95) but with ∼100-fold lower computation time. The tumor segmentation masks comparison between both methods exhibited significant overlap and shape similarity with high concordance CCC > 0.97 across measures. *Significance.* FAST-PET generated PET images with high quantitative accuracy comparable to GATE, making it ideal for applications requiring extensive PET image simulations such as virtual imaging trials, and the development and validation of image processing pipelines.

## 1. Introduction

Positron emission tomography (PET) is indispensable to patient management in oncology (Unterrainer *et al* 2020), neurology (Tai and Piccini 2004), and cardiology (Schofield *et al* 2021), offering unique insights into physiological or pathologic processes in both normal and diseased tissues. Recently, the trend in PET imaging has shifted from qualitative to quantitative analysis, driven by its potential to diagnose diseases, track therapeutic responses, and, ideally, predict prognosis and treatment response (Ziai *et al* 2016, Lammertsma 2017, Zaidi *et al* 2018, Paydary *et al* 2019). In this context, PET simulation, prized for its repeatability and capacity to establish a ground truth, emerges as an essential complement to clinical data, facilitating this shift toward quantitative analysis. It supports the accurate quantification of tracer uptake (Zhuang *et al* 2019), the development and validation of quantitative imaging workflow (Karakatsanis *et al* 2013, Samimi *et al* 2020), and advanced image processing pipelines (Rong *et al* 2015). Unlike physical phantoms, PET simulation offers unmatched control and flexibility in modeling tracer uptake distributions and facilitates studies without the need for scanner access, addressing limitations in the availability of real scanners.

PET simulation methods are broadly categorized into Monte Carlo (MC) simulations and analytical simulations. The MC technique meticulously simulates each positron emission and annihilation, photon transportation (including absorption and scattering), and detection occurring within the acquisition time by modeling the intricate underlying physics processes. By conducting multiple simulations for various positron emissions, MC simulation offers comprehensive insights into the imaging process (Buvat and Lazaro 2006, Paredes-Pacheco *et al* 2021). The strength of MC simulation lies in its detailed replication of physical processes, yielding highly accurate and realistic outcomes. Hence, MC simulation tools such as GEANT 4 Application for Tomographic Emission (GATE) (Jan *et al* 2004, Sarrut *et al* 2021), SIMSET (Haynor *et al* 1990), PET-SORTEO (Reilhac *et al* 2004, 2005), PeneloPET (España *et al* 2009) have been developed to facilitate research and development of new scanners and advanced image reconstruction techniques. However, this precision comes with the trade-off of high computational demand due to the stochastic sampling of individual particle tracks, especially when simulating voxelized patient geometries with the aim of achieving clinical image quality (IQ) level. This high computational burden prompted the development of analytical PET simulation methods that focus on the estimation of projection distribution while discarding detailed electron/photon tracking as a faster alternative (Thielemans *et al* 2012, Berthon *et al* 2015, Stute *et al* 2015, Häggström *et al* 2016, Pfaehler *et al* 2018). Such tools are valuable for the development and evaluation of quantitative PET imaging metrics and workflow as well as sophisticated image processing methods, such as segmentation methods. Existing analytical simulation methods exhibit shortcomings as they often fail to precisely replicate scanner geometries and detection formats and oversimplify scatter estimation in forward projections.

To address these issues, we propose an analytical simulation method: fast analytical simulator of tracer (FAST)-PET. In this work, FAST-PET is developed and validated as an accurate and efficient analytical PET simulation tool. The detailed workflow of FAST-PET is described in section 2.1. Its performance was first validated in the simulation of a National Electrical Manufacturers Association (NEMA) IQ phantom which is commonly used for assessing PET/CT IQ. We benchmarked FAST-PET simulated images against both experimental NEMA IQ phantom scan images and GATE simulations under different acquisition times in terms of their activity distribution and noise level. Since the NEMA IQ phantom falls short in replicating the heterogeneity and complex anatomy of real clinical patients, the effectiveness of FAST-PET is further validated with actual clinical data simulations which involved comparing intensity and texture features between FAST-PET and GATE simulations to establish quantitative similarity. Additionally, a segmentation task was adopted to compare tumor segmentation outcomes from both simulation methods. Collectively, FAST-PET was demonstrated to be a reliable and rapid analytical simulation method, producing images that closely match those from experimental scans and GATE simulations.

## 2. Method

### 2.1. Description of FAST-PET

FAST-PET is a PET analytical simulation and reconstruction method that is based on the Siemens reconstruction software *e7tools* (Siemens Healthineers, Knoxville TN). We previously developed a method for lesion insertion in brain simulations, used for PET quantitative accuracy validation (Hamdi *et al* 2021, 2023). The objective of FAST-PET is to create a tool capable of generating synthetic PET images that closely resemble those obtained from the actual scanner within an expedited timeframe (around 2 min for one clinical scan simulation, details in discussion section). This is achieved through precise forward projection, scatter, and random estimation that match the real scanner geometry and statistics. Although the same process should be able to apply to other Siemens scanner models, we focus on the development and validation of the simulation of the Siemens Biograph Vision-600 PET/CT scanner (referred to as ‘Vision-600 scanner’ hereafter) in this paper. FAST-PET can be accessed by contacting the corresponding author.

#### 2.1.1. Input and output

To define the subject of the simulation, FAST-PET requires a 3D activity and a 3D attenuation map which have the same size as the transaxial and axial field of view as the Vision-600 scanner: $72.6 \times 72.6 \times 26.2\,\,{\text{c}}{{\text{m}}^3}$. Variable acquisition durations may be allocated to determine the count levels in the simulation, thereby emulating distinct levels of noise. Users are free to choose the reconstruction settings such as the size of reconstructed images, post-reconstruction filter, and reconstruction algorithm that suit their needs. The output is the synthetic PET image in raw binary format with a header file documenting the metadata.

#### 2.1.2. Simulation process

**Figure 1.**
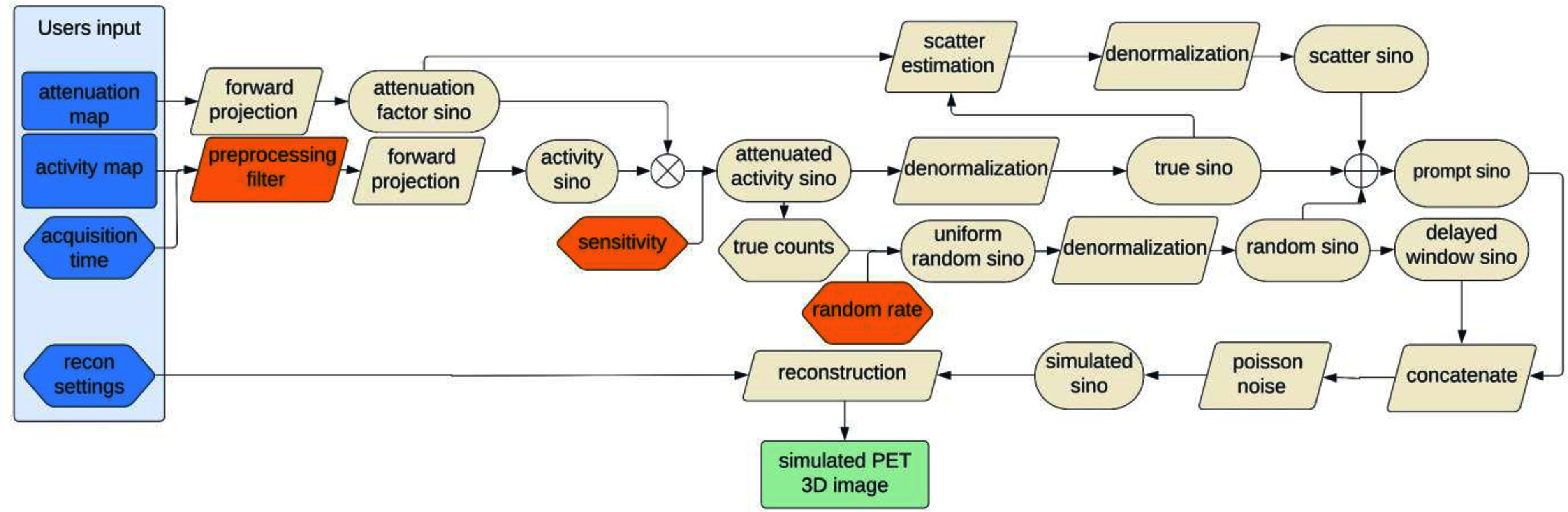
Pipeline of FAST-PET simulation. Blue blocks represent the input maps and settings from users and green block represents the output from FAST-PET. Orange blocks highlight steps that involve calibration.

Figure 1 presents a schematic overview of the FAST-PET simulation pipeline. It involves calibrating three parameters—preprocessing smoothing, sensitivity, and random rate—based on experimental data from the NEMA IQ phantom, with further details provided in section 2.2.1. FAST-PET then simulates PET images from the input activity and attenuation maps following these steps:

1. The input activity map is scaled to the size of $440 \times 440 \times 159$ ($1.65 \times 1.65 \times 1.646\,{\text{m}}{{\text{m}}^{\text{3}}}$ in *x*-, *y-, z*-direction) to make the size compatible with *e7tools* and multiplied with acquisition time to calculate the number of decays within each voxel and then smoothed by a 3D isotropic preprocessing Gaussian filter to simulate the intrinsic spatial resolution of PET imaging, for example, annihilation photon noncollinearity, positron range, camera resolution (Moses 2011).
2. The smoothed activity map is then forward projected into an activity sinogram of dimension $520\left( {\# \,of\,projections} \right) \times 50\left( {\# \,of\,views} \right) \times 815\left( {\# \,of\,planes} \right) \times 33\left( {\# \,of\,TOF\,bins} \right)$ using *e7tools* forward projector which follows Joseph’s method (Joseph 1982), mirroring the number of projections, angular mashing, axial data compression (span of 19) and TOF binnning used in the Vision-600 scanner following its geometry configuration.
3. The attenuation map is forward projected to an attenuation sinogram of dimension $520\left( {\# \,of\,projections} \right) \times 50\left( {\# \,of\,views} \right) \times 815\left( {\# \,of\,planes} \right)$ and then an exponential function is applied to it to obtain attenuation factor sinogram. The activity sinogram is attenuated by dividing the attenuation factor sinogram.
4. The sensitivity value is multiplied by the attenuated activity sinogram to scale the photon counts to the level of the real scanner. Then, summing up the attenuated activity sinogram gives the number of true events.
5. Random events are assumed to be uniformly distributed in the projection space (Boellaard *et al* 2004, Berthon *et al* 2015, Pfaehler *et al* 2018) and the random rate is defined as the fraction of simulated random counts over true counts. Then a uniform sinogram is scaled to the number of random counts for random simulation.
6. The scatter sinogram is estimated using the single scatter simulation algorithm (Watson 2007) in 2D mode and converted into 3D sinogram through the inverse single-slice rebinning algorithm (Lewitt *et al* 1994) via *e7tools*.
7. Since normalization will be applied in the image reconstruction step below (step 11), the simulated sinogram thus needs to be denormalized. The denormalization step is completed by dividing the normalization sinogram from the original sinogram to simulate geometric effects, crystal interference, and axial effect. The fully expanded compartment based normalization sinogram is created using *e7tools*. The attenuated activity sinogram, estimated scatter sinogram, and uniform random sinogram go through the denormalization step to create noise-free sinograms of true and random events.
8. The noise-free prompt sinogram is the summation of true, scatter, and random sinograms.
9. A delayed time window bin is calculated by the summation of the random sinogram along the TOF dimension and concatenated with the prompt sinogram for the purpose of random correction during the reconstruction process.
10. The final simulated sinogram is generated by applying Poisson noise element-wisely to the noise-free prompt and delayed window sinogram, simulating the photon arrival process as the Poisson process.
11. The simulated sinogram can be reconstructed using 3D ordinary Poisson ordered subset expectation maximization algorithm. The TOF reconstruction and the point spread function (PSF) correction are also applicable with parameters tuned for the Vision-600 scanner within *e7tools*. Other settings, such as different reconstruction sizes and post-reconstruction filters, can be specified by users too.

### 2.2. Calibration and validation with NEMA IQ phantom

FAST-PET was calibrated according to an experimental 10 minute scan of a NEMA IQ phantom obtained from the Vision-600 scanner. Then both experimental data and GATE simulation results were compared with FAST-PET across various acquisition times to validate the quantitative accuracy of FAST-PET.

#### 2.2.1. Phantom experiment and FAST-PET calibration

In the phantom experiment, the physical NEMA IQ phantom was placed at the center of the Vision-600 scanner’s imaging field of view and scanned for 10 min, gathering data in list mode format. The radionuclide used in the phantom was ^18^F. The standard NEMA IQ phantom contained six spheres with inner diameters of $10,\,13,\,17,\,22,\,28,\,37\,{\text{mm}}\,$(sphere 1–sphere 6) placed in a background volume of $9575\,{\text{ml}}$. The activity concentration in the background and spheres was $2930\,{\text{Bq}}\;{\text{m}}{{\text{l}}^{ - 1}}$ and $27\,\,369\,{\text{Bq}}\,{\text{m}}{{\text{l}}^{ - 1}}$ respectively, forming a lesion-to-background concentration ratio of $9.34:1$. For the purpose of FAST-PET calibration, the list-mode data was rebinned and reconstructed into one frame of size $440 \times 440 \times 159$ ($1.65 \times 1.65 \times 1.646\,{\text{m}}{{\text{m}}^{\text{3}}}$ in *x-, y-, z*-direction) using 3D OP-OSEM algorithm, with 8 iterations and 5 subsets, TOF, PSF correction, and no post-reconstruction filter.

In the FAST-PET simulation of the NEMA IQ phantom, the activity map was set to be the same as the experimental data, and the attenuation coefficient within the volume occupied by the NEMA IQ phantom was set to the same value as water $0.096\,{\text{c}}{{\text{m}}^{ - 1}}$ and other regions were set to zero. Initially, one sinogram of true events of a 10 min scan was generated by FAST-PET. The sensitivity was determined at $38\,{\text{cps}}/{\text{kBq}}$ by aligning the number of true events in the simulated sinogram with those in the experimental data, which was calculated by subtracting the estimated scatter and random events from all events detected. The random rate, calculated as 20%, was derived from the ratio of events in the delayed window bin to true events in the experimental data. Subsequently, a grid search on the full width at half maximum (FWHM) of the preprocessing Gaussian filter was conducted. After generating multiple 10 min simulated scans using the same reconstruction setting as in the experimental data with calibrated sensitivity and random rates but varying Gaussian filters, the FWHM of 3 mm was selected as it best matched the RCs within spheres on the reconstructed images.

#### 2.2.2. RC and coefficient of variation (COV) comparison

GATE simulation was performed on the Center for high performance computing (CHPC) platform employing a well-validated model of the Vision-600 scanner with phantom and source designed to emulate the NEMA IQ phantom in the experiment described above. A 10 min scan was simulated, and the data was acquired in a list mode format for further rebinning.

Five different count levels corresponding to 5 s (1.7 million counts), 10 s (3.5 million counts), 30 s (10.4 million counts), 60 s (20.7 million counts), 120 s (41.4 million counts) scans were considered in this study. For each count level, 5 images were reconstructed from the list mode experimental data and GATE simulated data and simulated using FAST-PET respectively as 5 independent noise realizations. The same reconstruction setting in section 2.2.1 was applied to all three cases across every noise level to produce comparable results.

The region of interest (ROI) of the background was defined as an annular-shaped area on 5 adjacent slices where no lesion appears while ROIs of lesions were selected to be sphere-shaped areas of the same size and position as inserted spheres. The RC was defined as the ratio of the observed to true activity within an ROI and measured for the quantification of the partial volume effect (Zhu *et al* 2018). For each ROI of sphere within the NEMA IQ phantom and noise level mentioned above, the mean of RCs over five noise realizations with standard deviation (STD) as the error bar were plotted as a function of sphere size and compared between images generated by the three methods. The COV, describing the variation in the intensity values within an ROI, was measured to quantify the noise level in the NEMA IQ phantom experiment and simulation. Under each noise level, COV was calculated within both ROIs of spheres and BG and averaged over 5 noise realizations. For each ROI, COVs of the three methods were plotted as a function of acquisition time and put together to facilitate comparison.

#### 2.2.3. Voxel-wise activity mean and STD distributions comparison

By calculating the voxel-wise mean and STD across 5 noise realizations, we generated the activity mean and STD maps to visualize the voxel-wise intensity distribution within PET images under high (5 s scan) and low (120 s scan) noise levels respectively. For each ROI of BG and spheres, histograms showcasing the intensity distributions of activity mean maps, generated from experimental NEMA IQ phantom images and GATE and FAST-PET simulated images, were plotted together according to the Freedman-Diaconis rule (Freedman and Diaconis 1981) for comparison. The same steps were followed to generate histograms for STD maps.

### 2.3. Validation with realistic clinical image simulation

Advances in quantitative PET imaging highlight the significance of intensity-based and texture-based feature analysis in aiding disease diagnosis (Punithavathy *et al* 2015, Moscoso *et al* 2018) and response prediction (Ypsilantis *et al* 2015, Nakajo *et al* 2017, Sun *et al* 2020). This points out the necessity to assess PET IQ in terms of multiple orders of features in real clinical images (Hatt *et al* 2017). In this section, we simulated clinical PET images using both FAST-PET and GATE, and comparisons were performed in terms of both intensity and texture-based features.

#### 2.3.1. Patient characteristics and acquisition protocol

The clinical assessments and scan data from 7 patients (mean age of $62.3 \pm 9.7$ years old, mean weight of $75.7 \pm 10$ kg) who had ^18^F-FDG PET scans were collected. Patients fasted for at least 4 h before the PET/CT scan and were injected with an FDG dose of approximately 555 MBq. Following a low-dose CT scan for attenuation correction and anatomic correction, a single whole-body PET acquisition was performed, utilizing continuous bed motion, at approximately 60 min post-injection. All PET images were reconstructed using the 3D OP-OSEM algorithm per manufacturer-recommended parameters: point-spread function correction with TOF; 4 iterations, 5 subsets, $440 \times 440$ matrix ($1.65 \times 1.65 \times 4{\text{ m}}{{\text{m}}^{\text{3}}}$ in *x-, y-, z*-direction), all-pass filter with scatter, random, decay, and attenuation correction.

#### 2.3.2. FAST-PET and GATE simulation

Patient PET/CT images were cropped from the lung to the upper abdomen, matching the axial field of view of the Vision-600 scanner (26 cm). Attenuation maps were derived from CT images using the same method employed by GATE (Jan *et al* 2004) to ensure comparability between simulation results. To accommodate for the extensive time and computational resources required by GATE voxelized phantom simulation, the original PET images and attenuation maps were resized to $220 \times 220 \times 53$ matrix ($3.3 \times 3.3 \times 4.94\,{\text{m}}{{\text{m}}^{\text{3}}}$ in *x-, y-, z*-direction) and served as the input activity and attenuation maps. The same input images were used in FAST-PET to maintain consistency across both simulation methods. For each patient, one 5 min PET acquisition was simulated and reconstructed into a matrix of the same size as input maps using the 3D OP-OSEM algorithm with TOF; 4 iterations and 5 subsets, all-pass filter with point-spread function, scatter, random and attenuation correction in both simulation methods.

#### 2.3.3. Features analysis

Two kinds of features, intensity-based features, and texture-based features, were calculated and compared between FAST-PET and GATE. Among intensity-based features, peak (the mean intensity in a 1 cm^3^ spherical volume centered on the voxel with the maximum intensity level within the ROI), mean and median intensity were used to quantify the activity level within organs and tumors. Skewness (Groeneveld and Meeden 1984) and COV were adopted to measure the asymmetry of the activity distribution and the intensity variability within ROIs. For texture-based features calculations, the original images were discretized using a fixed bin width of 100 (kBq ml^−1^), and grey level co-occurrence matrices (GLCM) were generated for $0^\circ $, $45^\circ $, $90^\circ $ and $135^\circ $ directions (Hall-Beyer 2000). Five features, joint average, joint entropy, energy (Angular second moment), homogeneity (inverse difference), and correlation, were derived from the GLCM of each direction, and the average over four directions was taken as the final feature values (Zwanenburg *et al* 2020). For each patient, features analysis was performed on six normal organs (spleen, liver, stomach, pancreas, left and right lung) and tumors. MOOSE segmentation (Isensee *et al* 2021, Sundar *et al* 2022) was applied to CT images to segment organs and generate ROIs. Tumors were segmented manually on the original patient PET images and excluded from masks of normal organs if they were inside organs. In total, 50 ROIs (42 for organs and 8 for tumors) were generated and applied to both FAST-PET and GATE simulated images in feature calculations. Linear regression and its 95% confidence intervals (Zou *et al* 2003) along with Pearson correlation coefficients (Cohen *et al* 2009) were calculated between features of FAST-PET and GATE simulated images to compare the activity distributions and texture patterns in PET images generated using both simulation tools.

### 2.4. Segmentation task-oriented comparison

Beyond quantitative analyses of intensity distributions and texture patterns, task-based evaluation is crucial for its capacity to correlate metrics with real-world applications (Xu *et al* 2015, Dutta *et al* 2023). The segmentation task, pivotal in clinical image analysis as a precursor to feature extraction, demands significant attention in evaluating the quality of simulated PET images. It offers unique perspectives on human observers’ image interpretation and diagnostic quality of the image. In this section, we compared FAST-PET and GATE through the lens of tumor segmentation results derived from images simulated by these two methods.

Three experienced medical image analysts segmented tumors in both GATE and FAST-PET simulated images. The simultaneous truth and performance level estimation (STAPLE) algorithm (Warfield *et al* 2004) was applied to generate consensus maps from three independent segmentations to mitigate inter-observer variability (Dutta *et al* 2021), which is referred to as ‘STAPLE segmentation’ for brevity. The spatial conformity of STAPLE segmentation results was validated by calculating the overlap-based and volume-based segmentation evaluation metrics as well as in terms of agreement of shape-based features. The overlap-based metrics (Taha and Hanbury 2015) including the Dice similarity coefficient (DSC) (Dice 1945), true positive rate (TPR), precision, and Matthews correlation coefficient (MCC) (Chicco *et al* 2021) were applied to quantify the similarity of the segmentation by comparing the area of overlap while the volume-based metric, volumetric similarity (VS) (Taha and Hanbury 2015), was used to measure the volume difference between two segmentations. Moreover, the volume, surface area, major axis length, minor axis length, and least axis length (Breckon and Solomon 2011) of tumor masks were adopted as shape-based features to describe the overall shape of segmented tumors. The concordance between shape metrics derived from STAPLE segmentations on FAST-PET and GATE simulated images was evaluated using Lin’s concordance correlation coefficient (CCC) (Lawrence and Lin 1989) to examine their shape similarity.

## 3. Results

### 3.1. Validation with NEMA IQ phantom experiment and simulation

**Figure 2.**
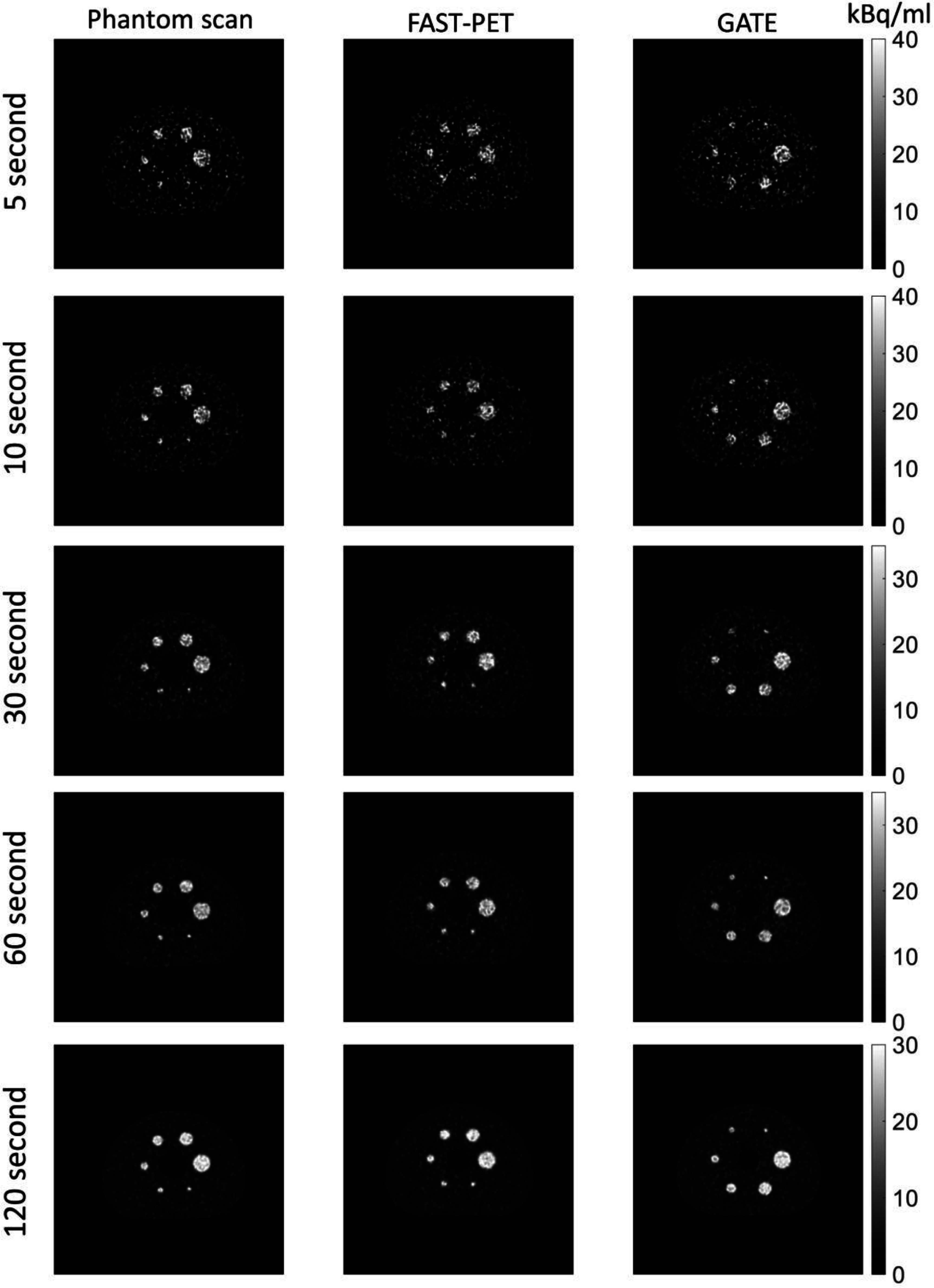
Representative slices of phantom scan images (first column), FAST-PET (second column), and GATE (third column) simulated images under 5 s, 10 s, 30 s, 60 s, and 120 s scan time.

Figure 2 displays a representative slice of experimental images, FAST-PET, and GATE simulated images of NEMA IQ phantom under five noise levels: 5 s, 10 s, 30 s, 60 s, and 120 s scan time. All slices are zoomed in to enhance the visualization of regions containing the NEMA phantom.

#### 3.1.1. Comparison of recovery coefficient and COV

**Figure 3.**
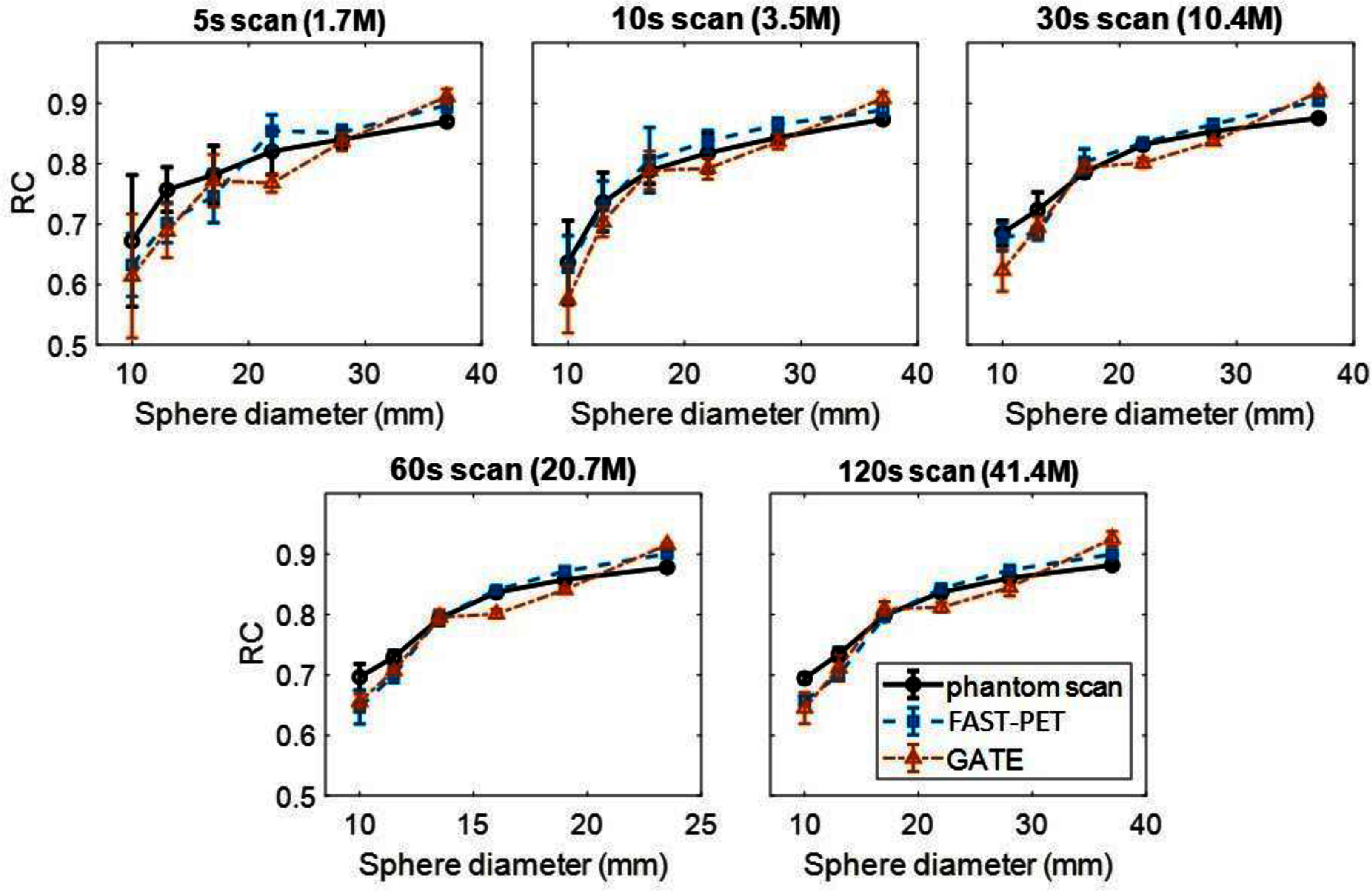
Means and standard deviations of recovery coefficient (RC) of 6 inserted spheres over 5 noise realizations under 5 different noise levels: 5 s frames (1.7 million counts), 10 s (3.5 million counts), 30 s (10.4 million counts), 60 s (20.7 million counts), 120 s (41.4 million counts).

Figure 3 displays the relationship between the mean of RC and sphere diameter across five noise levels (5 s, 10 s, 30 s, 60 s, 120 s), including the STD as error bars. In all three cases, the mean of RC rises as the sphere diameter increases, aligning with the expectation that the partial volume effect is more pronounced in small spheres. Additionally, the STD increases with decreasing sphere diameter or acquisition time, indicating that higher noise levels and fewer pixels within the ROI amplify RC value fluctuation. For both FAST-PET and GATE simulations, the relative error of the mean RC remains under 10% for all spheres and within 6.5% for the four larger spheres across all noise levels. As demonstrated, there are no significant differences in terms of RC between FAST-PET simulated images, GATE simulated images, and experimental physical phantom images at the five noise levels.

**Figure 4.**
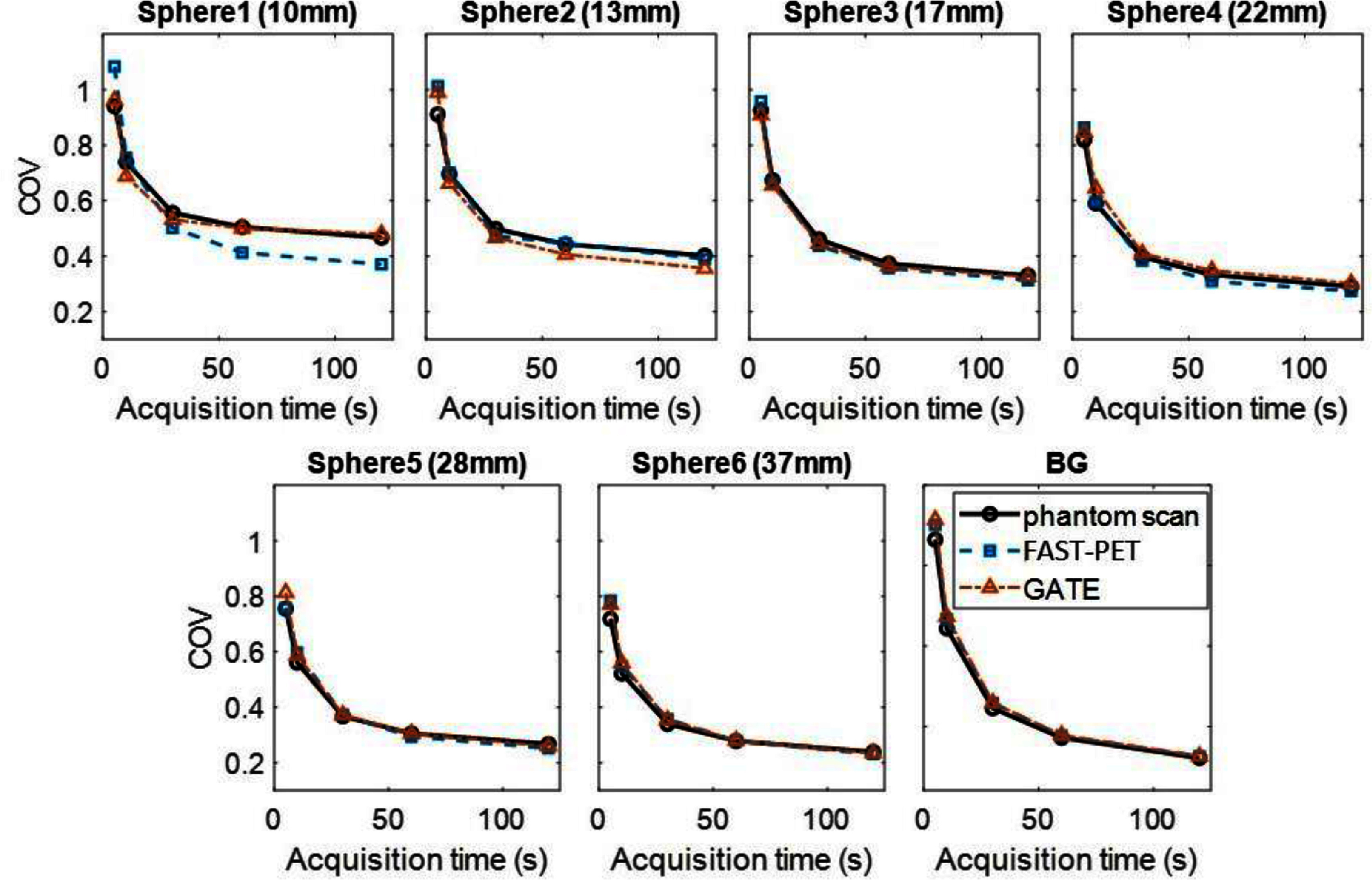
Coefficients of variation (COV) within inserted spheres and background versus acquisition time.

Figure 4 illustrates COV as a function of acquisition time for ROIs of six spheres and BG, depicting a consistent trend across the three images where COV decreases as acquisition time increases. Since the BG area is not affected by the partial volume effect, COV within the BG ROI accurately reflects the noise level. The relative bias in COV of FAST-PET and GATE images compared with experimental data stays within 6% and 8% in the BG ROI across all noise levels respectively. Apart from the two smallest spheres, COV values from both simulated images closely match experimental data, with relative bias staying below 10% at every noise level. However, simulations yield lower COV values for the smallest spheres compared to experimental data, likely due to simulation challenges in accurately replicating the PET system’s resolution.

#### 3.1.2. Comparison of voxel-wise activity mean and STD distribution

**Figure 5.**
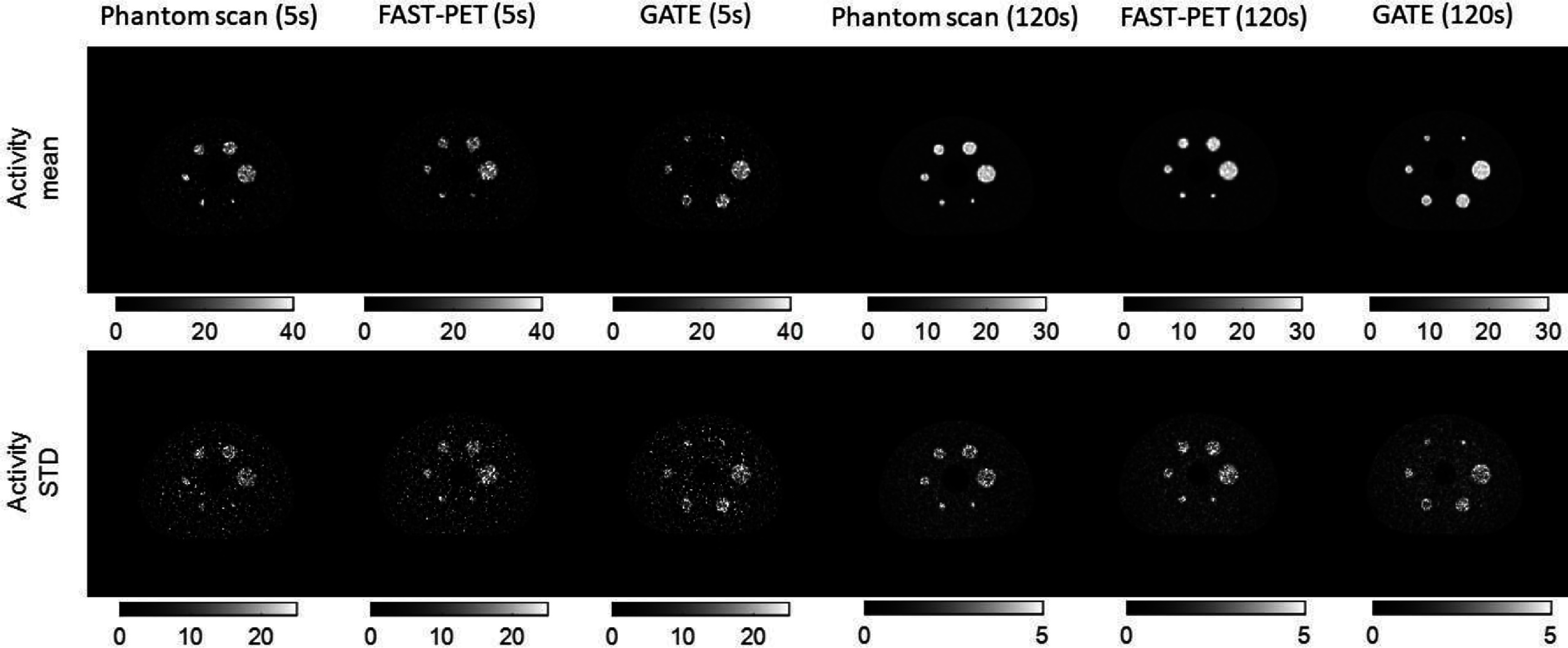
Representative slices of (first row) voxel-wise activity mean maps and (second row) voxel-wise activity standard deviation maps (STD) of experimental images, FAST-PET, and GATE simulated images under 5 s scan time and 120 s scan time.

**Figure 6.**
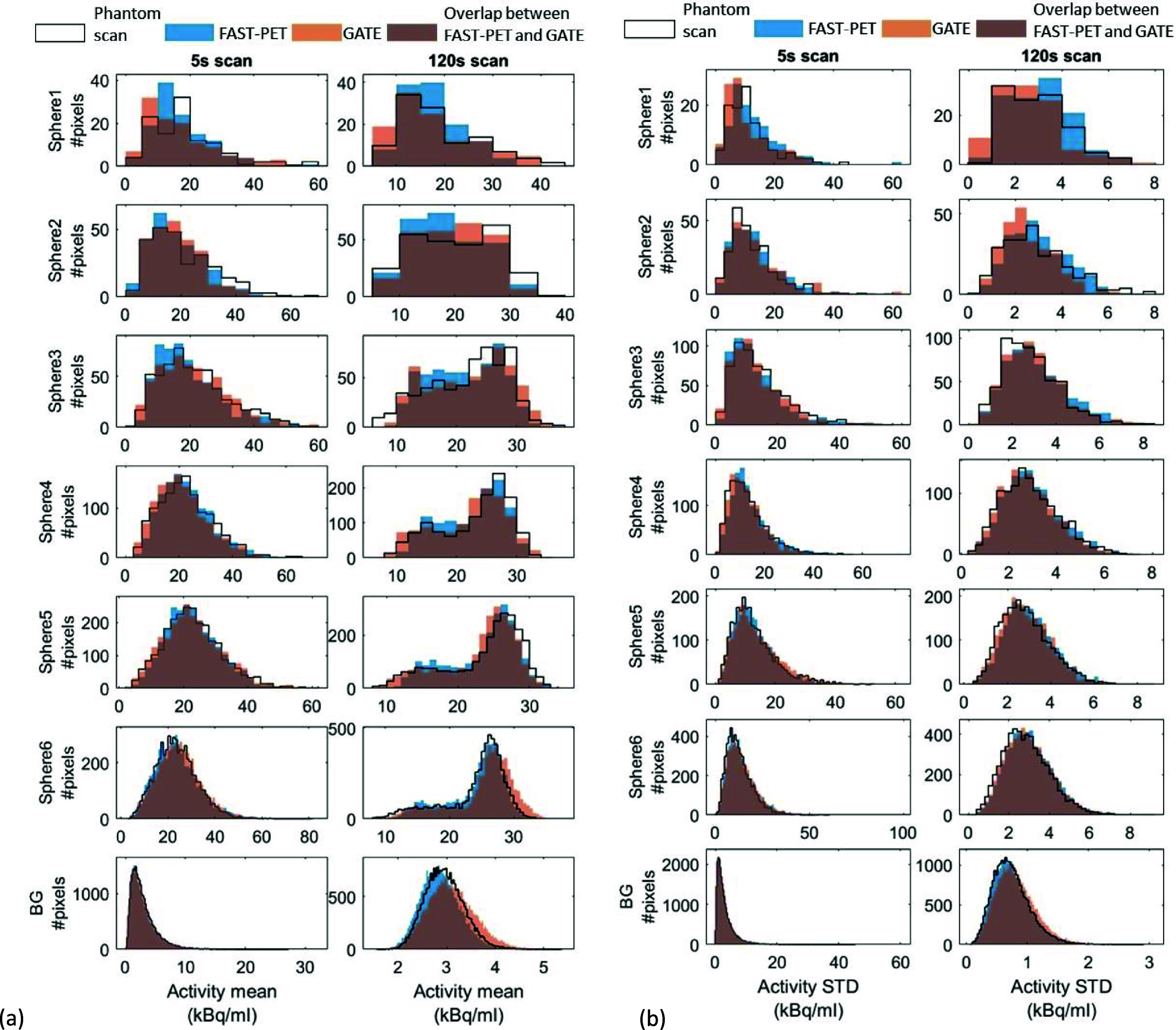
Distributions of (a) activity mean and (b) activity standard deviation (STD) within regions of interest of inserted spheres and background of phantom scan images, FAST-PET, and GATE simulated images under 5 s scan time and 120 s scan time.

Representative slices of the activity mean and STD maps of high (5 s scan) and low (120 s scan) noise images from the three methods are presented in figure 5. Figure 6 shows the intensity distribution of activity mean and STD within ROIs of spheres and BG at both acquisition times. The intensity distribution of the activity mean map within the BG ROI is skewed to the left in the 5 s scans and becomes nearly symmetric in the 120 s scans. This distribution is consistent with the expected log-normal distribution of pixel values within PET images reconstructed using the expectation-maximization algorithm (Barrett *et al* 1994). In low-noise images, the intensity distribution of the activity mean map within spheres skews to the right, highlighting the partial volume effect where peripheral pixels in spheres show lower activities than assigned values. Because of low photon counts, the activity STD in the 5 s frame is markedly higher and more broadly distributed than that in the 120 s frame. Across both acquisition times, the intensity distributions of activity mean and STD maps of the FAST-PET and GATE simulated images and experimental images demonstrate considerable similarity, suggesting that images produced by both simulation methods closely resemble real scan images in terms of quantitative characteristics across various noise levels.

### 3.2. Validation with realistic clinical image simulation

**Figure 7.**
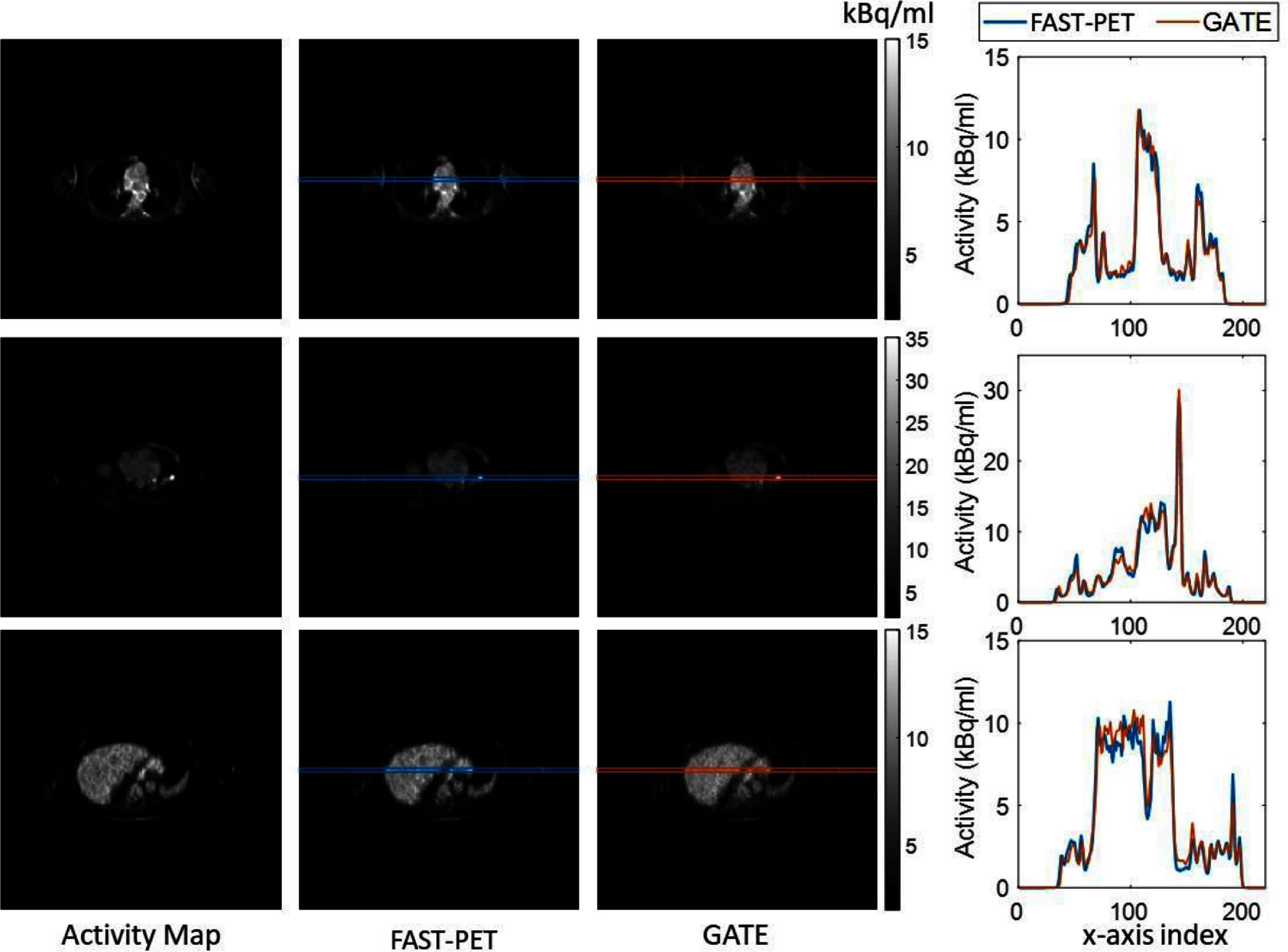
Representative slices of activity maps and simulated images from FAST-PET and GATE in clinical patient simulations. The last column shows profiles within the rectangular of both simulated images along the *x*-axis.

Figure 7 presents representative slices of activity maps used for simulation along with simulated images generated by FAST-PET and GATE from three patients. A 3 pixel-high rectangle positioned at the vertical center and spanning from left to right was drawn on each simulated image. The final column of figure 7 displays profiles from both simulated images within this rectangle along the *x*-axis, by averaging values of the 3 pixels sharing the same *x*-coordinate. The overlap of these profiles confirms FAST-PET’s capability to create synthetic PET images comparable to those from GATE simulations in a clinical imaging context.

**Figure 8.**
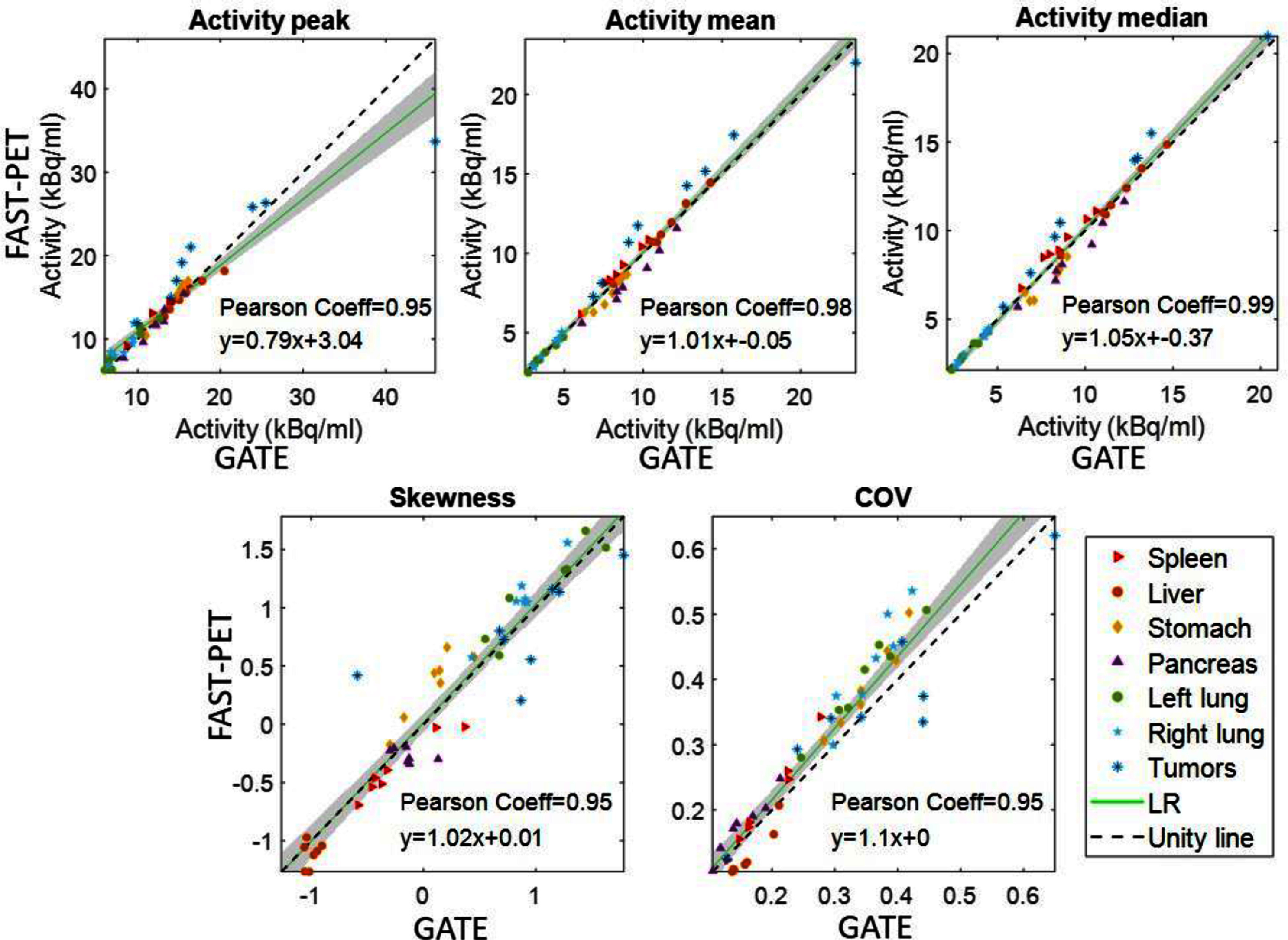
Scatter plots comparison of intensity-based features between FAST-PET and GATE simulated images.

**Figure 9.**
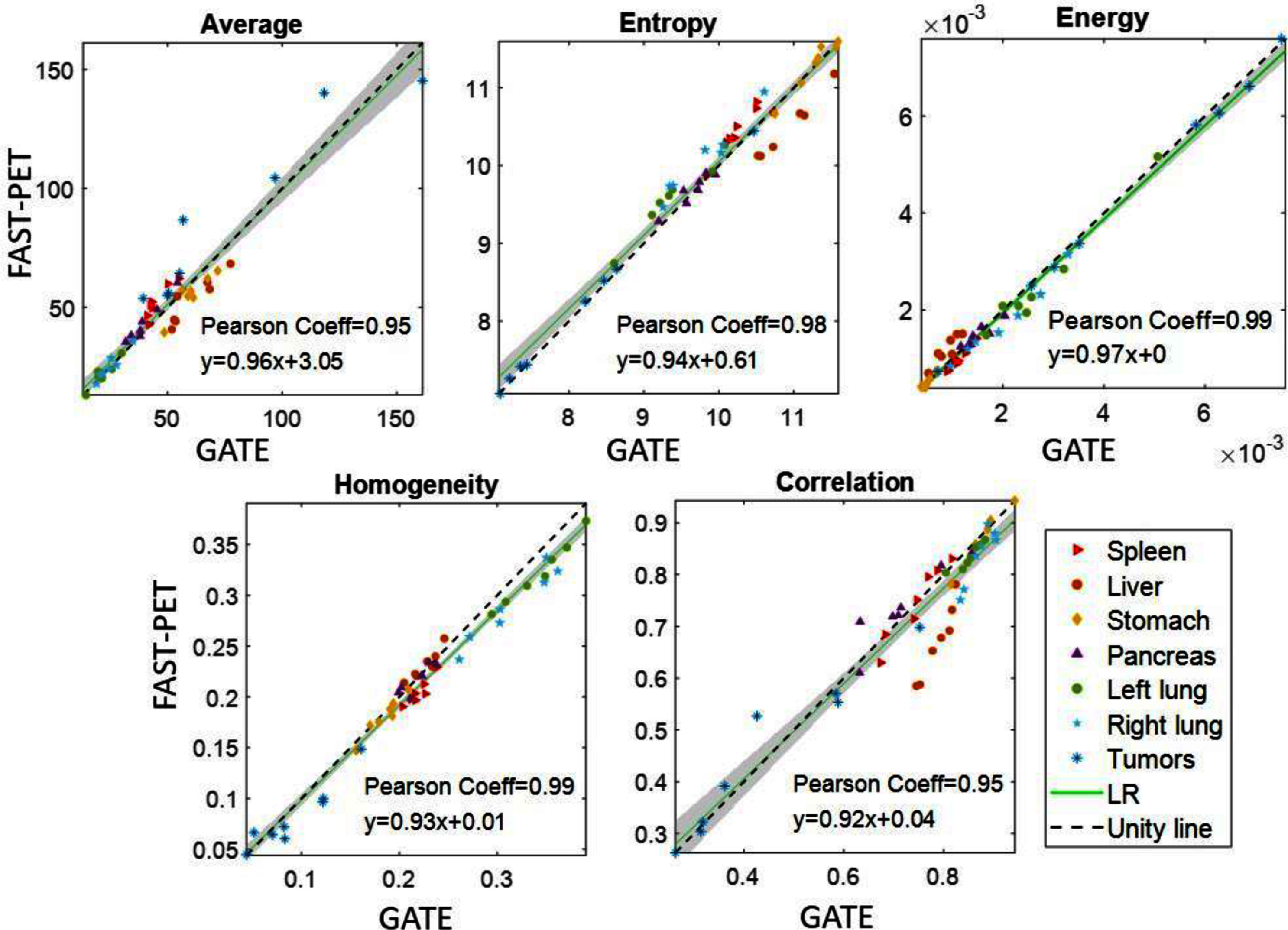
Scatter plots comparison of texture-based features between FAST-PET and GATE simulated images.

The intensity-based and texture-based features measured from FAST-PET simulated images are compared with those from GATE simulations. Figures 8 and 9 illustrate the correlation of features from both sets of simulated images across all ROIs of organs and tumors. In terms of activity peak and mean, FAST-PET exhibits a median relative difference of 3.4% and 0.19% compared with GATE respectively. The slopes of most regression curves are close to 1, suggesting low discrepancy in measured features between FAST-PET and GATE simulated images (details about regression results are in tables S1 and S2 in the supplementary file) and the high values of Pearson correlation coefficients indicate there existed strong correlations.

### 3.3. Segmentation task-oriented comparison

**Figure 10.**
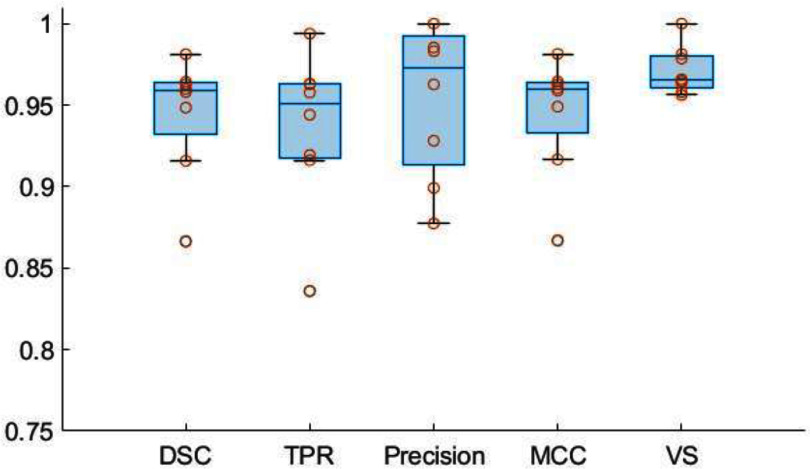
Box plots of metrics evaluating the tumor segmentation similarity between FAST-PET and GATE simulated images: Dice similarity coefficient (DSC), true positive rate (TPR), precision, Matthews correlation coefficient (MCC), and volumetric similarity (VS). The closer the metrics approach 1, the greater the similarity between the two segmentations.

Figure 10 features box plots of five different metrics evaluating the similarity between STAPLE segmentation masks on FAST-PET and GATE simulated images for eight tumors. The median values of DSC, TPR, precision, MCC, and VS are 0.96, 0.95, 0.97, 0.96, and 0.97 respectively, indicating a high degree of overlapping between segmentations from FAST-PET and GATE images with similar tumor volume. Additionally, one sample showed lower values for DSC, TPR, and MCC (around 0.85), due to the small size of the tumor, where minor misalignments can notably affect the overlap-based metrics. CCC values of shape-based features in table 1 are above 0.97, further validating the shape congruence between the two segmentations. Overall, segmentation masks from both simulations closely match, demonstrating comparable performance in segmentation tasks across both simulated images.

**Table 1.** Concordance correlation coefficients of shape-based features between tumor segmentations on FAST-PET and GATE simulated images.

Volume	Surface area	Major axis length	Minor axis length	Least axis length
0.997	0.998	0.970	0.983	0.994

## 4. Discussion

In this work, we developed and validated FAST-PET, a fast analytical method to simulate PET images. FAST-PET simulated NEMA IQ phantom images are quantitatively comparable to experimental images and GATE simulations in terms of RC, noise level, and voxel-wise activity distribution. Furthermore, FAST-PET produces similar results as GATE in simulating clinical images, as demonstrated by the high agreement in intensity-based and texture-based features. A segmentation task-oriented evaluation further demonstrates that tumor masks generated from FAST-PET images closely match those from GATE, endorsing FAST-PET’s utility in task-based assessment. While FAST-PET was developed in conjunction with e7 tools, the methodology employed in developing FAST-PET can be applied to other scanners. Additionally, we are planning on developing FAST-PET into an online PET simulation platform to make it publicly accessible, allowing users to simulate PET images for their own research purposes. Also, FAST-PET can be accessed by contacting the corresponding author. This dual approach ensures that FAST-PET is widely available to both independent researchers and collaborative projects.

GATE, as a state-of-the-art MC-based simulation tool, provides users with the utmost flexibility, allowing for the selection of source type, detailed definition of system geometry down to the crystal level, digitizer customization, and so on. While its meticulous electron/annihilation photon tracking modeling ensures high fidelity to the physical decay process, it demands significant computational resources and time. Efforts to mitigate this issue have involved GPU acceleration (Lai *et al* 2019, Galve *et al* 2024, Herraiz *et al* 2024), but this may introduce new barriers due to GPU requirements and management. The time required for voxelized source simulations via GATE increases not only as the number of counts increases but also as the granularity of the source becomes finer. As demonstrated in section 2.3, 5 min clinical ^18^FDG-PET frames were simulated using voxelized phantoms with voxel size $3.3 \times 3.3 \times 4.94\,{\text{m}}{{\text{m}}^{\text{3}}}$. GATE simulations were executed in parallel on the CHPC platform across 500 CPU cores, including model types Intel Xeon Gold 6226R, Intel Xeon Gold 6240L, and Intel Xeon Gold 6226, and required approximately 3 h per patient simulation (around 1500 h on a single CPU core). In contrast, FAST-PET demonstrates a marked improvement in efficiency, completing one patient simulation in around 110 s on a single Intel Xeon W-1290P processor: forward projection, scatter and random estimation, Poisson noise generation, and final reconstruction take around 5, 25, 50, 30 s respectively. Notably, the simulation time for FAST-PET remains consistent at about 110 s, regardless of the complexity of the input data, whether for NEMA IQ phantom or clinical image simulations. Due to the gridding limits of e7tools, FAST-PET is limited to simulating features down to 1.65 mm since FAST-PET must resize the input map to meet the input restriction of e7tools, which can result in a loss of information during the resizing process. Overall, FAST-PET consistently produces images quantitatively similar to those from GATE simulations, as demonstrated through the NEMA IQ phantom and clinical image simulations.

Compared with other analytical simulation tools, SMART (SiMulAtion and ReconsTruction) PET, PETSTEP (PET Simulator of Tracers via Emission Projection), and STIR (Software for Tomographic Image Reconstruction), FAST-PET outperforms in terms of both accuracy and efficiency. According to the literature (Berthon *et al* 2015, Pfaehler *et al* 2018, Khateri *et al* 2019), SMART-PET simulation ranges from 3–15 min, PETSTEP 4–10 min, and STIR around 4–6 h per PET image simulation. In terms of forward projection accuracy, FAST-PET is uniquely tailored for the target scanner type, ensuring high fidelity by performing forward projection adhering to specific scanner geometry, generating sinograms mirroring actual scanner formats, and configuring sensitivity and random rates based on experimental data. Conversely, SMART-PET controls system noise and resolution through only one scale factor and a pre-projection Gaussian filter, while TOF is not implemented in the forward projection of PETSTEP. In terms of scatter estimation, FAST-PET employs a model-based algorithm offering more accuracy than SMART-PET and PETSTEP, which approximate scatter sinograms by forward projecting the blurred input activity map. STIR allows a flexible definition of the scanner geometry and thus supports precise forward projection, however at the same time this requires additional efforts from users to establish a comprehensive modeling of the target scanner. Thus, overall, FAST-PET has numerous advantages over available analytical simulation methods.

The need for numerous realistic simulated PET images is prevalent in various domains, including virtual imaging trials. The conventional trials, despite being the gold standard for testing medical interventions, grapple with safety and ethics issues, patient recruitment challenges, and a lack of comprehensiveness due to the under-representation of rare subpopulations (Fan *et al* 2022, Cullen *et al* 2023). Consequently, this has given rise to the development of in silico trials, which solve the problems above but require extensive image simulations to include a comprehensive representation of subject characteristics in a statistically significant population (Abadi *et al* 2020, Badano *et al* 2023) and call for an efficient PET simulation tool to conduct virtual imaging trials with minimum time and resource expenditure. Moreover, simulated PET images serve as a crucial element for the development and validation of advanced image processing as well as quantitative and computational pipelines such as image segmentation, denoising, and kinetic analysis due to their ability to provide known ground truth (Le Maitre *et al* 2009, Dutta *et al* 2022). Testing of novel algorithms demands a large number of test images to provide clinically relevant and robust results considering variations in tissue geometries and patient uptake distributions. Furthermore, recent advances in deep-learning and artificial intelligence technologies further signify the need for realistic simulated images for training of the network. Therefore, FAST-PET can potentially be used as a platform for generating extensive datasets of realistic PET images to support the development and validation of advanced PET image analysis algorithms.

A forthcoming development direction for FAST-PET involves expanding its compatibility with various scanner models. While this paper primarily focuses on establishing a simulation pipeline for Siemens Biograph Vision-600 PET/CT images using *e7tools*, the intention is for this pipeline to be easily adaptable for simulating images from other scanners with minor modifications. Additionally, FAST-PET employs an isotropic Gaussian filter for preprocessing smoothing, yet PET system resolution varies spatially (Budinger 1998, Rahmim *et al* 2013). Further work will explore investigating the modeling of spatial variation of the system resolution especially on the deterioration of resolution radially from the tomograph axis. Moreover, the fixed random rate in FAST-PET does not fully reflect the variability seen in actual PET imaging, which can be influenced by factors such as activity level. Enhancing the accuracy of random rate modeling to more accurately mirror real-world conditions will be a critical area of development. Besides this, we are exploring more efficient sampling algorithms to reduce the time consumed by noise sampling, currently the most time-intensive stage of our simulations. Finally, ongoing efforts will be dedicated to incorporating continuous bed movement in the simulation, aiming to fully replicate all functions of the scanner.

## 5. Conclusion

FAST-PET was developed and validated as a fast, and accurate analytical PET image simulation method. It performs precise forward projection, scatter, random estimation, and reconstruction following the real scanner geometry and statistics, thereby generating highly realistic PET images. We have shown that FAST-PET allows the fast generation of PET images (within 2 min) and reproduces simulated PET images that are quantitatively comparable to experimental images and GATE simulations. Furthermore, we have demonstrated that images simulated by FAST-PET yield comparable outcomes in task-oriented segmentation evaluations to those by GATE, highlighting its utility in developing and assessing PET segmentation algorithms. The high accuracy and efficiency of FAST-PET affirm its capacity to simulate complex and heterogeneous tracer uptake with quality akin to GATE, making it well-suited for scenarios requiring extensive PET image simulations.

## Data Availability

All data that support the findings of this study are included within the article (and any supplementary information files).
